# THE RELATIONSHIP BETWEEN SEDENTARY BEHAVIOR AND COGNITIVE DOMAINS AMONG PEOPLE LIVING WITH DEMENTIA

**DOI:** 10.1093/geroni/igad104.3656

**Published:** 2023-12-21

**Authors:** Charmi Patel, Stevie Lewis, Deborah Jehu

**Affiliations:** Augusta University, Augusta, Georgia, United States; Augusta University, Augusta, Georgia, United States; Augusta University, Augusta, Georgia, United States

## Abstract

Greater sedentary behavior negatively impacts cognition among older adults without dementia; however, the relationship between sedentary behavior and cognitive domains among people living with dementia (PWD) is unclear. Our aim was to explore the association between sedentary behavior and cognitive domains in PWD in residential care facilities. The testing took place at one nursing home (n=15/23), one memory care unit (n=4/23), and one assisted living facility (n=4/23). Participants completed a battery of cognitive tests (global cognition: Montreal Cognitive Assessment; executive function: Trail Making test, Digit Span Backwards Test; perception and orientation: Benton Judgment of Line Orientation Test; language: Boston Naming Test; learning and memory: Rey Auditory Verbal Learning Test; complex attention: Digit Symbol Substitution Test). We used the Morse Fall Scale to measure fall risk. Participants wore an actigraphy monitor on their wrist over seven days to measure sedentary behavior. Most participants were male (74%), white (87%), and had unspecified dementia (48%). Participants were sedentary for 8.0±2.7 hours/day. The regression model for sedentary behavior was significant (R2=0.82, F(10, 12)=5.39, RMSE=92.47, p< 0.05). Greater sedentary behavior was associated with poorer language function (β=-0.82, p=0.02), poorer learning and memory (β=-0.82, p=0.002), and greater fall risk (β=-0.43, p=0.03). Sedentary behavior was not associated with the other cognitive domains. Greater sedentary behavior may indicate a decline in language function, learning and memory, and increase fall risk in PWD. Sedentary behavior should be considered in routine assessments, as it may play an important role in monitoring cognitive decline in PWD in residential care facilities.

